# Altering workplace attitudes for resident education (A.W.A.R.E.): discovering solutions for medical resident bullying through literature review

**DOI:** 10.1186/s12909-016-0639-8

**Published:** 2016-04-27

**Authors:** Heather B. Leisy, Meleha Ahmad

**Affiliations:** Department of Ophthalmology, New York University School of Medicine, 462 First Avenue – NBV 5N18, New York, NY 10016 USA

**Keywords:** Bullying, Medical training, Residency, Resident harassment, Mistreatment

## Abstract

**Background:**

Physicians-in-training are challenged every day with grueling academic requirements, job strain, and patient safety concerns. Residency shapes the skills and values that will percolate to patient care and professional character. Unfortunately, impediments to the educational process due to medical resident mistreatment by bullying remain highly prevalent in training today.

**Methods:**

A PubMed literature review was undertaken using key terms to help define resident mistreatment by bullying, determine its prevalence, identify its potential causes and sequelae, and find suggestions for changing this detrimental culture of medical training.

**Results:**

We identified 62 relevant articles. The most frequently noted form of mistreatment was verbal abuse, with the most common perpetrators being fellow physicians of higher hierarchical power. Mistreatment exists due to its cyclical nature and the existing culture of medical training. These disruptive behaviors affect the wellbeing of both medical residents and patients.

**Conclusions:**

This article highlights the importance of creating systems that educate physicians-in-training about professional mistreatment by bullying and the imperative in recognizing and correcting these abuses. Resident bullying leads to increased resident stress, decreased resident wellbeing as well as risks to patient safety and increased healthcare costs. Solutions include education of healthcare team members, committee creation, regulation of feedback, and creation of a zero-tolerance policy focused on the health of both patients and residents. Altering workplace attitudes will diminish the detrimental effects that bullying has on resident training.

## Background

There are several studies and a few reviews discussing the prevalence of bullying of medical trainees. However, no review to date focuses on why this problem exists or how we might find solutions to reduce workplace harassment of residents. Only after we fully understand the root cause of this problem can focus be given to implementing methods of resolution.

Key characteristics of resident mistreatment are articulated throughout the literature. These include repeated intimidating, undermining, or distressing behavior directed at a victim, who usually holds less power. Several definitions of bullying, a key component in resident mistreatment, are shown below:

*“Bullying is repeated acts and practices that are directed at one or more workers, which are unwanted by the victim, which may be done deliberately or unconsciously, but clearly cause humiliation, offense, and distress, and may interfere with job performance and/or cause unpleasant working environments* [1]*”.*

*“Bullying may be seen as something that someone repeatedly does or says to gain power and dominance over another, including any action or implied action, such as threats, intended to cause fear and distress…The behavior has to be repeated on more than one occasion and there must be evidence that those involved intended or felt fear* [2]*”.*

*“[Bullying is] any behavior which is humiliating, abusive (physically or psychologically) or serves to enforce who is ‘in’ or ‘out’ of the group. It can range from objects being thrown at people to condescension, or berating in front of peers or privately* [3]*”.*

Some specific examples or signs of bullying include the following: *“*persistent attempts to belittle and undermine your work, persistent unjustified criticism and monitoring of your work, persistent attempts to humiliate you in front of colleagues, intimidatory use of discipline/competence procedures, undermining your personal integrity, destructive innuendo and sarcasm, verbal and non-verbal threats, making inappropriate jokes about you, persistent teasing, physical violence, violence to property, withholding necessary information from you, freezing out/ignoring/excluding, unreasonable refusal of applications for leave/training/promotion, undue pressure to produce work, setting of impossible deadlines, shifting goalposts without telling you, constant undervaluing of your efforts, persistent attempts to demoralize you, removal of areas of responsibility without consultation or discrimination on ground of race or gender [4].” Additional examples or signs of bullying include unexplained rages, non-verbal communication such as eye rolling, constant alteration of work targets or circulating negative rumor [2].

Many of these behaviors, especially if chronic, can be classified as psychological harassment [5]. In the workplace, these behaviors often occur in three distinct phases: 1) “antilocation”, characterized by prejudicial gossip restricted to a small “in-group” circle and “behind the back” of the victim, 2) avoidance of the victim by the crowd and associates, and 3) open harassment of the victim including discrimination, alienation, exclusion, offensive remarks and jokes. The third phase, which we most often associate with bullying, may result in the victim either being expelled from the working environment or, in severe cases, attempting suicide [1].

Rates of workplace bullying in medical training are high throughout the world (Table 1). A study amongst medical residents in the United States showed that during their training, 69.8 % have experienced workplace abuse [6]. Similar statistics have been mentioned in other studies in North America [7, 8]. In a study of United Kingdom (UK) residents, it was reported that 37 % self-identified as having been bullied and 84 % of these residents had experienced one or more bullying behaviors [4]. Cyberbullying, an emerging method of bullying through technology such as text message and email, was seen to occur in almost half of medical residents at a UK institution [9]. In a study of family medical residents in Canada, 45 % reported experiencing a form of intimidation, harassment, and/or discrimination during their training, and over half had experienced this behavior more than once [10]. Similar rates were reported in Ireland, South Australia, New Zealand and other regions of Canada [2, 11–15]. Reported rates of medical trainee mistreatment are even higher in Asia and Africa, ranging from 77 % in Nigeria to 97 % in Oman [16–23]. The majority of these studies have found that main source of inappropriate behavior, harassment, and belittlement of physicians-in-training is from their fellow physicians in superior positions [6, 10, 16, 21, 24, 25]. The most common form of abuse in medical training is verbal [8, 10, 16, 17, 20, 26] and these comments are commonly belittling, undermining, or humiliating [10, 21].

**Table 1:** Global prevalence of medical resident bullying

First Author, Year	Design	Country of conduct	Subjects	Terminology	Prevalence^a^	Key perpetrators^a^
Shinsako [6]	Cross-sectional	United States	Medical Residents	Generalized workplace abuse	70 %	Percentage unspecified; perpetrators within training program
Nabi 2013 [11]	Cross-sectional	South Australia	Junior Medical Officers on Surgical Night Shifts	Bullying	54 %	Percentage unspecified; senior surgeons and ED staff
Scott 2008 [12]	Cross-sectional	New Zealand	House Officers, Registrars	Bullying	50 %	Consultants and nurses in equal frequency
Farley 2015 [9]	Cross-sectional	United Kingdom	First and second year trainee doctors	Cyberbullying	46 %	26 % by consultants, 35 % by other trainees
Quine 2002 [4]	Cross-sectional	United Kingdom	House officers to Senior Registrars	Bullying	84 %	Percentage unspecified; peers, senior staff, managers
Crutcher 2011 [10]	Retro-spective	Canada	Family medicine Graduates	Intimidation, harassment and/or discrimination	45 %	71 % by specialist physicians
Cohen 2005 [13]	Cross-sectional	Canada	Resident physicians	Intimidation or harassment	51 %	42 % staff physicians
Cheema 2005 [15]	Cross-sectional	Ireland	Junior Doctors	Bullying	30 %	61 % by senior physicians (EU & non-EU averaged)
Ogunsemi 2010 [18]	Cross-sectional	Nigeria	Residents in training	Intimidation and harassment	78 %	30 % from other residents, 7 % from consultant staff
Nagata 2009 [16]	Cross-sectional	Japan	Residents	Mistreatment	85 %	35 % physicians
Bairy 2007 [19]	Cross-sectional	India	House officers and postgraduate students	Bullying	89 %	15 % from medical personnel
Al-Shafaee 2013 [17]	Cross-sectional	Oman	First year medical residents	Verbal/ academic abuse	88 %	98 % of verbal abuse from consultants/ specialists
Fnais 2013 [20]	Cross-sectional	Saudi Arabia	Medical Residents	Harassment/ discrimination	84 %	Percentage unspecified; majority consultants
Imran 2010 [21]	Cross-sectional	Pakistan	Junior Doctors	Bullying	64 %	52 % consultants
Ahmer 2009 [22]	Cross-sectional	Pakistan	Postgraduate psychiatric trainees	Bullying	80 %	Percentage unspecified; consultants most likely perpetrators

^a^ All percentages are rounded off to nearest integer

## Methods

**Fig. 1:**
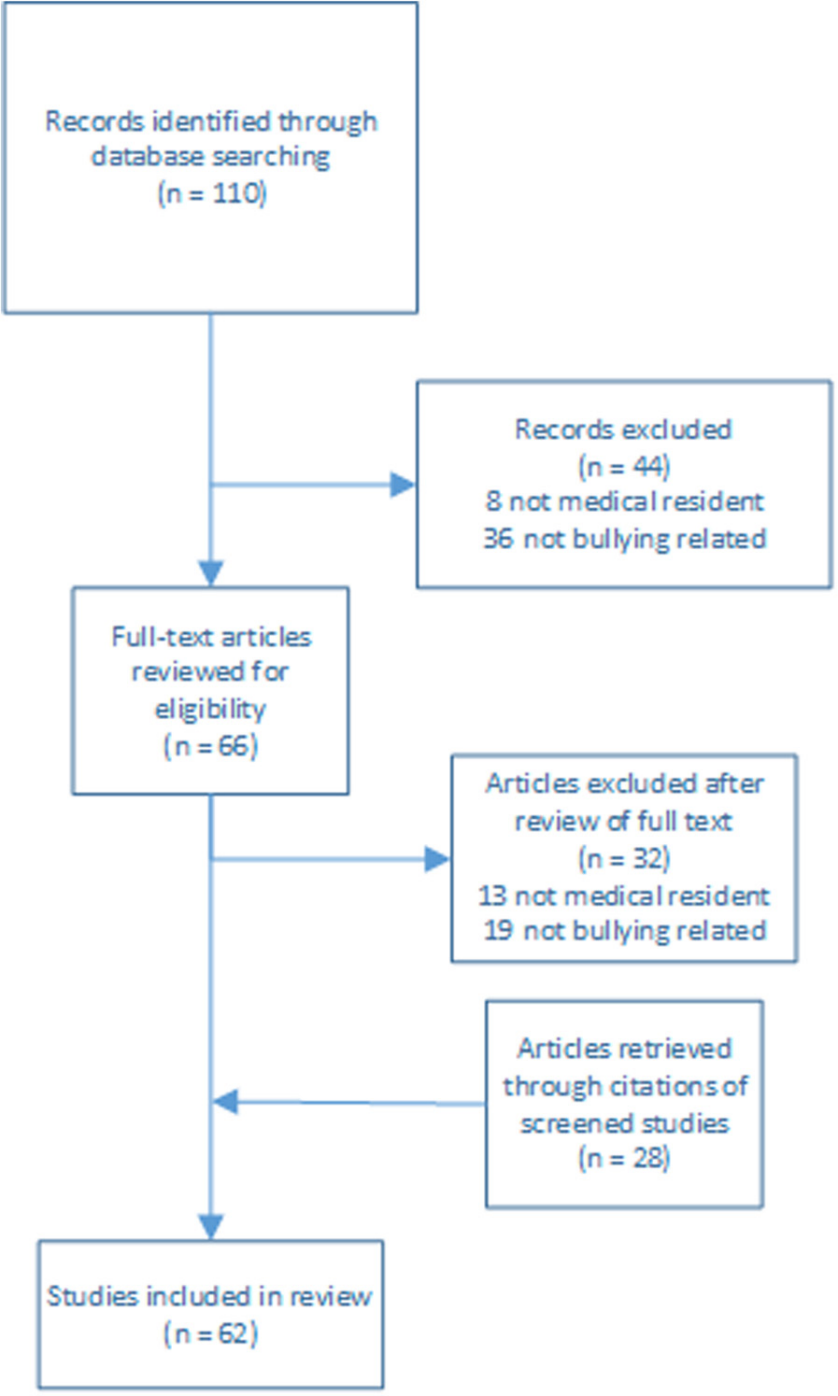
Flowchart of review selection process and results

Relevant articles on the topic of medical resident mistreatment by bullying were identified by searching with related Medical Subject Headings (MeSH) and text words in PubMed (January 1, 1999 to March 4, 2016). The MeSH terms included “bullying” and “internship and residency” with various forms of the text terms: “resident”, “residency”, “junior doctor”, “resident physician”, “doctor in training”, “intern”, “house officer”, “intern”, “mistreatment”, “harassment”, intimidation”, “bully”, “belittlement”, “humiliation”, “disrespect”, “demean”. Titles and abstracts of these articles obtained from the database searches were reviewed to ensure that they pertained to medical training mistreatment. Articles falling outside of the searched date range, not written in English, or not pertaining to resident medical training were excluded. The search process yielded 34 articles with an additional 28 articles obtained through examination of reference lists (Fig. 1). Information from these 62 articles were extracted.

## Results

### Causes of resident mistreatment: key themes

In researching the potential causes for resident mistreatment, a number of themes appeared, including hierarchy, silence, incognizance, fear, acceptance/denial and a legacy of abuse. Each theme will be discussed in the following sections.

#### Hierarchy

Hierarchy, or power differences, has been cited as the underlying cause of the bullying relationship that often leads to workplace mistreatment [1], even in residency [10]. A study in the French judicial system showed that over 90 % of people convicted of workplace harassment were of higher hierarchical power than the victim [27]. Hospitals have a particularly hierarchical foundation in which bullying can flourish [2, 17, 28]. The hierarchal culture of healthcare affects the attitudes, values, and behaviors of medical trainees [29]. A survey on bullying found that “most of the negative behaviors were perpetrated by other doctors, in a pecking order of seniority [30].” The Joint Commission, in its review of bullying, stated, “intimidating and disruptive behaviors are often manifested by health care professionals in positions of power [31]”.

#### Silence

Although a hierarchical system sets the stage for resident mistreatment, it is silence that allows mistreatment to continue in our medical training systems without opposition. Silence exists not only due to poor communication but also because of the decision to choose silence over the risk of repercussions in reporting.

The culture of silence in healthcare was discussed in the article “Silence Kills: The Seven Crucial Conversations in Healthcare,” which underscores the difficulty healthcare workers face in effectively communicating topics of disrespect, poor teamwork, and lack of support. They state that “more than half of the healthcare workers surveyed have witnessed a small percentage of their coworkers break rules, make mistakes, fail to support, demonstrate incompetence, show poor teamwork, disrespect them, and micromanage,” however, “less than one in ten say anything about it [32]”.

Other sources also expose a troubling culture of silence in medical resident mistreatment and bullying [2, 16, 31], one of which found that of those residents who experienced abuse only 12 % reported the abuse to a supervisor [16]. The Joint Commission stated, “40 % of clinicians have kept quiet or remained passive during patient care events rather than question a known intimidator [31]”.

#### Incognizance

Naiveté on what constitutes an abusive behavior and how to report it can perpetuate mistreatment. Studies show that residents often do not report an incident of abuse because they are unaware of what qualifies as abuse [16, 17], they rationalize intimidation and harassment as being a “functional educational tool [33],” or they lack knowledge on how and to whom they should report the incident [2, 5, 16, 17, 30]. Studies analyzing resident knowledge of the reporting process showed that only about half knew the process required to report such mistreatment [10, 13].

Medical training supervisors have also demonstrated a lack of education on how to address bullying and its consequences [28]. In an article “Creating a Culture of Mutual Respect,” the authors state that many hospital policies address overt mistreatment such as discrimination, criminal activity, sexual assault or physical abuse but that grey areas such as bullying and intimidation still need to be addressed [34].

#### Fear

Resident fear of retaliation and a lack of confidence in our healthcare systems to adequately and appropriately handle reports of mistreatment can also contribute to flourishing mistreatment [35]. Fear of retaliation has been widely shown to be a reason for the persistence of resident mistreatment [10, 17, 21, 30, 31, 34]. In many cases, the resident does not report an incident of abuse due to the fear that it would negatively influence his or her career, the fear of labeling, or the fear that greater mistreatment would occur if reported [16]. Residents may fail to report witnessed mistreatment as a way of “protecting their own learning experience,” or to avoid “sticking out [36].” A survey-based study showed that 79 % of doctors who experienced bullying were too afraid to complain [2, 12].

Additionally, many sources state that residents often do not believe that the policies and process of reporting will exhibit a just application and outcome [16, 34]. In one study, only 44 % of residents felt that the process of reporting abuses would be fair and adequate [13], setting the stage for a culture in which mistreatment can go unchecked.

#### Acceptance/denial

In many cases, the denial that bullying even exists in medical training forms a lattice on which it can flourish [2]. The culture of medicine is one of “professional dominance” in which systems and subjects are rarely subject to enquiry [37]. Articles discussing this issue showed that bullying occurs when it is part of the accepted culture of an organization [8, 28]. With the acceptance of an atmosphere of professional dominance, rather than one of countervailing powers [38], trainees are often not aware that disruptive and inappropriate actions actually constitute abuse. A review cited that the trainees experiencing humiliation and criticism often thought that these were part of normal training [5]. An example of this is “pimping,” which had long been considered an acceptable variant of the Socratic method but is now coming under scrutiny as, in certain forms, it can result in learner mistreatment through humiliation [39, 40].

#### Legacy of abuse

Mistreatment can persist as a taught behavior, creating a cycle or legacy of abuse. An article analyzing abuse occurring to first year residents commented that the legacy of bullying within the medical profession may be because “abuse begets abuse [17].” It is known that medical trainees assimilate their mentors’ behaviors and attitudes, so when exposed to abusive behaviors, these actions may be perpetuated [29]. Abusive behaviors are not confined to a small number of individuals [31], thus an overarching systemic problem must be occurring. When medical trainees are affected, the patient can even become the direct victim of this abuse cycle [10]. Potentiating risk factors for falling prey to the cycle of abuse are stress and low self-esteem [28, 31].

### Sequelae of resident mistreatment: individual and systemic effects

Resident bullying is detrimental to the victim and his or her family as well as the healthcare system as a whole. These disruptive behaviors can lead to medical errors, harming of patients, extra costs to a hospital system, and dissolution of quality care. Given the importance of workplace attitudes, the Joint Commission made the following sentinel alert:

*“..Intimidating and disruptive behaviors can foster medical errors, contribute to poor patient satisfaction and to preventable adverse outcomes, increase the cost of care, and cause qualified clinicians, administrators and managers to seek new positions in more professional environments. Safety and quality of patient care is dependent on teamwork, communication, and a collaborative work environment. To assure quality and to promote a culture of safety, health care organizations must address the problem of behaviors that threaten the performance of the health care team* [31]*”.*

Here we discuss potential areas that are compromised with resident mistreatment through bullying, focusing on both potential victims: the medical resident and the patient.

#### Effects on the individual

When a resident is bullied, this negative behavior is detrimental to their emotional health [10, 16, 30, 41]. Physicians-in-training who have experienced belittlement and harassment during their training experience poor mental health including depression, stress, low self-confidence, and suicidal ideation [2, 6, 17]. A commentary on the experience of Canadian physicians stated:

*“Students and junior doctors experience uncivil workplace behaviors such as bullying, belittlement and racism, creating a toxic environment. These acts have far-reaching negative effects on personal health, personal relations, career satisfaction, and create a ripple effect that permeates every facet of the health system… Healthy workplace initiatives are needed as residents and medical students indicate that medical training has adversely affected their health* [42]*”.*

Resident depression and stress has been linked to a higher rate of burnout [43, 44]. Burnout, described as “emotional exhaustion, in which overwhelming work demands deplete the individual’s energy”, “depersonalization and cynicism, in which the individual detaches from the job,” and “feelings of inefficacy, in which the individual perceives a lack of personal achievement [43],” has been linked with workplace bullying [45]. Resident mistreatment has been shown to promote a decreased satisfaction with residency [8, 10, 16], and can even be associated with thoughts of desertion [18, 30]. Burnout can even cause these residents to rethink their career choice of medicine altogether. One study showed that over 20 % of residents would not pursue medicine again if given the chance to relive their career [13, 20], and several would even advise others not to become physicians [17].

Some have suggested that increased pressure and intimidation serves to sharpen a resident’s skills or determination to be a physician, and as such, is a rite of passage. However, survey-based research has shown that only 2 % of medical doctors who experienced abuse agreed that it “increased eagerness and indomitable determination to learn medicine [16].” In actuality, residents most often experience anger, diminished eagerness to work, depression, increased feelings of difficulty at work, health problems, and thoughts of dropping out in response to intimidation [16, 46].

Bullying causes other damaging psychological effects such as increased stress [10, 30, 47]. Studies have related the psychological stress reaction in those who are bullied to symptoms similar to post-traumatic stress disorder [27, 28, 45]. Additionally, the social and emotional difficulties caused by bullying can have negative effects on residents’ home lives [15, 47]. Trainees, victimized by hierarchy, are often made to complete personal tasks for senior physicians such as shopping or baby-sitting [5]; additional strain in the work domain will lead to fewer resources to fulfill one’s role for this at home [47]. Loss of familial support systems that provide stress relief and happiness [47] can promote physicians-in-training to seek out other forms of stress-relief which are potentially more dangerous. Studies have found increased alcohol consumption, cigarette smoking, and drug usage in those residents faced with increased stress and mistreatment [18, 48].

Bullying, especially when it causes increased work-home interference, may lead to a loss of professionalism on the part of the resident. Stress and burnout are associated with unprofessional behavior [44, 47], which can affect healthcare organizations as a whole. Trainees, when exposed to unethical behavior such as bullying, feel an obligation to join in themselves for fear of poor evaluations and not fitting in with the team [29]. Therefore, it’s best for the workplace to remove any source of bullying before it festers throughout the organization.

#### Patient safety, medical errors and quality of care

Bullying, and its associated behaviors, has proven risks for patient safety. A study found 67 % of those witnessing disruptive behavior felt that this behavior contributed to adverse patient events and 27 % felt that it contributed to patient mortality [49]. Another study showed that almost half of surgical team members who had witnessed disruptive behavior by attending surgeons were aware of adverse events resulting from this behavior [50]. The article, “Creating a Culture of Mutual Respect,” states that “rude, abusive, or intimidating behavior can hamper the ability to provide effective and safe patient care [34].” Organizations that encourage their members to suppress effective communication and teamwork by promoting fear or ridicule hold increased risks to patient safety and litigation [38, 46, 51]. Direct patient mistreatment may occur by providers who experience mistreatment during their training, as described in *Legacy of Abuse*. Creation of a work environment in which team members feel safe to address patient safety concerns are recommended for best practices [51].

The breakdown in effective communication secondary to bullying can lead to medical errors [52]. The Joint Commission cited that 70 % of unexpected medical deaths or serious injuries were caused by communication failures [34]. The vastness of medical errors resulting from communication gaps were also analyzed in the article, “Silence Kills: The Seven Crucial Conversations in Healthcare,” which showed that “more than 60 % of medication errors are caused by mistakes in interpersonal communication [32],” resulting in over 195,000 deaths per year in U.S. hospitals. When lapses in communication were investigated, they found the underlying problem to be different forms of bullying in many cases. The article states, “one in five physicians said they have seen harm come to patients as a result of these concerns [coworkers breaking rules, showing poor teamwork, showing disrespect to others] [32].” They argue for open and healthy conversation as it would lead to increased productivity, decreased turnover, and decreased medical errors [32].

Furthermore, other effects of resident bullying, like stress and depression, can promote medical errors. Depressed residents make more medical errors [47], such as six times more medication errors than their colleagues [10], and are the main source of medical error reporting [47]. Stress has been associated with medical errors and poor patient care [42, 47]. In fact, stress management courses decrease the rate of malpractice claims and medication errors [42].

Finally, bullying decreases quality of care by affecting individual job performance and an organization’s financial bottom-line [28, 46]. Costs can occur from replacement training of those individuals who left the organization, from medical errors, and from litigation for lapses in patient safety. Less funds generated towards re-investment into facilities, research, or care along with decreased job performance ultimately lead to lower quality of patient care. Additionally, bullying impairs quality because it ultimately robs an organization of their most diligent, dedicated, and competent workers [51]. This individual becomes the scapegoat and gets pushed out of an organization for problems that are actually deep-seated institutional deficiencies [51].

## Discussion

Analysis of the causes and sequelae of resident mistreatment through bullying allows for the development of resolution methods. Outlined below are several approaches that can be applied to healthcare training systems to reduce bullying behaviors.

Education, in several areas, is one means by which bullying can be minimized. Awareness of what entails workplace abuse and how to report it are the first steps [5, 7, 17]. Next, residents must be aware of available well being resources [13]. Training modules in emotional intelligence [53], communication [47, 49], leadership skills [2, 34], assertiveness training [49], work-life balance [51], empathy [51], burnout [47], conflict management [49] and stress relief [44, 47] can be incorporated into the resident curriculum to resist mistreatment.

Emphasizing team-based care at all levels of training can also combat bullying. Lucian Leape Institute’s “Recommendation 6” emphasizes the importance of teaching medical trainees to work in interdisciplinary teams as a core competency [29]. The present deficiency of this training results in lack of team care, which results in adverse health outcomes [29]. A team-based mentality to medicine reduces its hierarchical nature and has the potential to improve outcomes [34, 54] by using the strength and knowledge of the group to improve care [3, 51].

Good leadership by physicians in supervising roles prevents bullying even by a simple presence. Inadequate, biased, and unsupportive supervision have been correlated with stress, burnout, and poor mental health in resident physicians [44, 47]. Therefore, the leaders of the department, especially the program directors, must be physicians who can be supportive, impartial, and available. Review of the program director with “regular input from the residents and faculty is critical in this process, and an annual faculty retreat and a regular residency advisory council with appropriate resident input might be one such forum in which to conduct a reevaluation [44].” Leadership is crucial in defining the culture of an organization and they should create one that is just and safe [55].

Leadership should also create a culture of prioritizing academics in which the patients comes first but emphasis of care is in teaching, not just providing service [29, 44]. In a qualitative study, internal medicine residents identified having a “mean attending” as the most important of 40 detractors from having successful attending rounds [56]. A focus on teaching will give respect and needed attention to residents by leading physicians, creating an atmosphere accepting of feedback and new ways of thinking [51]. The atmosphere should allow mindful awareness of ones capabilities, encouraging seeking of help without the implication of weakness [51].

Programs that focus on inter-physician support could provide a solution to bullying [42]. Encouraging collegiality and supportive relationships mitigate stress and increase worker retention [42]. As mentioned previously, supervisors are crucial in creation of the training atmosphere. One of the easiest ways to form a supportive system is through mentoring of physicians-in-training. Good mentoring supports residents and reduces the rates of harassment and belittlement experienced during medical training [6, 44]. The very basis for mentoring echoes back to the original Hippocratic Oath of “my colleagues will be my brother (sister), teach and be taught [42]”.

Confidential mental health services should be readily accessible to residents to combat bullying. Medical trainees with a history of depression are more likely to be targets of abuse [41]. A recent review articled showed the prevalence of depression and depressive symptoms to be 21 to 43 % in medical residents [57]. Colleagues, program directors, external psychiatrists and psychologists can be sources of support for medical residents [13, 47]. Creation of a family-friendly work environment with special emphasis from the supervisor in implementing this atmosphere is needed [47]. Provision of counseling and psychological services have been successful in helping residents navigate their medical training [5, 42].

Standardization and restructuring of the learning environment, especially any “hidden curriculum” (resident interactions, hierarchical abuse, patient dehumanization, cultural expectations of overt personal sacrifices for career), is required [25, 47]. Residents must be involved in the development of the curriculum and policies on harassment [47], identifying potentiating factors like stress and addressing them [34]. Feedback standardization can decrease favoritism and ever-changing standards. “Perceptions of Stress” states, “once a standardized system is established, the residents’ duties, roles, and responsibilities will be clear, helping in standardized, objective, and fair evaluation [44]”.

Creation of committees for mediation and investigation of bullying reports have been recommended as a solution to bullying [34, 35, 49]. In his article, “Mistreatment of Students and Residents: Why Can’t We Just be Nice?”, Dr. David Sklar suggests that these committees “think of every incident of mistreatment as possibly predictable and potentially preventable,” rather than thinking of this as an unmanageable problem [58]. The Maimonides Medical Center has been a model for putting in place this type of committee, and when allegations were reviewed, 50 % of perpetrators were in violation of the code of conduct and 40 % were told to be mindful of their behaviors [34], thus demonstrating the need for such a committee. If a full committee cannot be formed, regular resident forums can be used to develop optimal communication and improve workplace environments [44].

In conjunction with creation of review committee, reporting methods must be further developed. First, a definitive protocol for reporting mistreatment and bullying must be developed [5]. Second, a safe mechanism for reporting that relieves the fear of retaliation is needed [17, 30, 49]. Third, the attitude towards bullying must be of zero tolerance [2, 29]. The Joint Commission supports creation of a zero-tolerance system for combating bullying through: education of all team members on professional behavior, code of conduct, zero tolerance in lapses of this code, use of non-retaliation clauses in the code, definition of the process of disciplinary action, provision of interpersonal education to leaders, establishment of awareness of the detrimental effects of bullying, creation of systems to monitor for unprofessional behavior, and creation of a committee to review and mediate any complaints [31].

In the worst-case scenario, if a program cannot change, there must be exit strategies for residents being mistreated. Post-graduate training must increase the opportunities and clarify the mechanism for changing programs in residency [13]. This will afford those who are harassed the ability to leave their program while also allowing those who have decreased satisfaction in their career choice a chance to assure their wellbeing by performing more satisfying work either in another specialty or profession altogether.

Opinions for resolving mistreatment from a resident, a medical education advocacy institute, and an expert are outlined below. Each, in their own way, emphasize the importance of changing attitudes, ensuring patient safety, and creating a zero tolerance system as key actions in bullying prevention.

A resident perspective on how to solve the problem of conflict and mistreatment in residency suggested the following: checking [your] ego at the door, respecting each team member for the skills and training they bring to the table, listening to one another—especially when someone voices concern, keeping in mind that patient safety is our number one priority, speaking up no matter how uncomfortable this may seem [59].

The Lucian Leape Institute for medical education advocacy suggests the following solution: “Recommendation 1: Medical school and teaching hospital leaders should place the highest priority on creating learning cultures that emphasize patient safety, model professionalism, enhance collaborative behavior, encourage transparency, and value the individual leader; eliminate hierarchical authority gradients that intimidate others and stifle teamwork; demonstrate non-tolerance for abusive or demeaning behaviors; enforce a zero tolerance policy for confirmed egregious disrespectful or abusive behaviors [29]”.

An expert in the field of advocacy against bullying, outlines the following mistreatment assessment process: develop standards of acceptable behavior for your organization; determine the monitoring unit for the organization for these accepted standards; establish the process of reviewing grievances; form a committee to review grievances; determine penalties for violations to these standards; communicate these above organization changes highlighting a zero-tolerance attitude [28]”.

From all perspectives, the issue of residency bullying needs to be resolved. Below is our summary of recommended changes:

1. Educate residents and attending physicians on what qualifies as bullying and the consequences of these actions. Provide training modules that emphasize lacking attributes such as leadership, assertiveness, communication, emotional intelligence, conflict resolution.
2. Educate residents and other providers on how to report instances of harassment within your institution. Specifically, residents should be given copies of publicly-available resources from the Accreditation Council for Graduate Medical Education (ACGME) such as “Procedures for Addressing Complaints and Concerns against Residency/ Fellowship Programs and Sponsoring Institutions [60],” “Institutional Requirements for Resident/ Fellow Learning and Working Environment [61], ” and “Distinguishing Between Concerns and Formal Complaints [62]”.
3. Creation of an anonymous reporting system and committee for reviewing of complaints, free of retaliation. This committee should determine acceptable behavior for the organization and penalties if not followed.
4. Standardization of training feedback to residents with accurate and timely filing methods. Additionally, residents should be required to provide feedback on the program and training staff in a standardized, accurate, anonymous, and timely fashion.
5. Creation of a culture focused on patient safety, academics, team-based care, and the wellbeing of the organization’s members, while advocating a zero-tolerance policy on bullying.
6. Promotion and advertisement of resident support programs such as mentoring and confidential mental health care services.
7. Increased flexibility in training and exit strategies for those who have experienced mistreatment.

## Conclusion

Awareness of bullying in residency training is critical for both resident and patient health. Through understanding the definition, prevalence, causes, and effects of this type of disruptive behavior, we can begin to stop it.

### Ethics

Not Applicable.

### Consent to publish

Not Applicable.

### Availability of data

Journals found on publicly available repositories.

## Abbreviations

A.W.A.R.E.: altering workplace attitudes for resident education
UK: United Kingdom
MeSH: Medical Subject Headings
ACGME: Accreditation Council for Graduate Medical Education
